# How to Tailor Porous Boron Nitride Properties for Applications in Interfacial Processes

**DOI:** 10.1021/accountsmr.2c00148

**Published:** 2023-01-30

**Authors:** Ioanna Itskou, Anouk L’Hermitte, Sofia Marchesini, Tian Tian, Camille Petit

**Affiliations:** † Barrer Centre, Department of Chemical Engineering, Imperial College London, LondonSW7 2AZ, United Kingdom; ‡ Department of Materials, Imperial College London, LondonSW7 2AZ, United Kingdom; § National Physical Laboratory, Hampton Road, TeddingtonTW11 0LW, United Kingdom

## Abstract

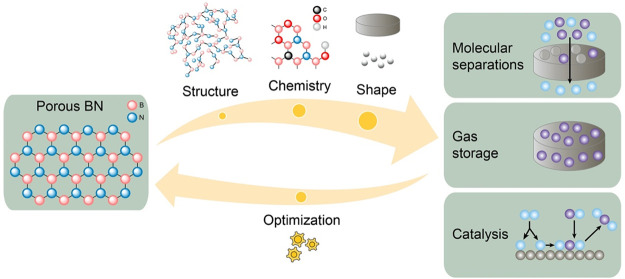

The research of new porous materials for applications in interfacial processes is key to addressing global energy and sustainability challenges. For example, porous materials can be used to store fuels such as hydrogen or methane or to separate chemical mixtures reducing the energy currently required by thermal separation processes. Their catalytic properties can be exploited to convert adsorbed molecules into valuable or less hazardous chemicals, thereby reducing energy consumption or pollutants emissions. Porous boron nitride (BN) has appeared as a promising material for applications in molecular separations, gas storage, and catalysis owing to its high surface area and thermal stability, as well as its tunable physical properties and chemistry.

However, the production of porous BN is still limited to the laboratory scale, and its formation mechanism, as well as ways to control porosity and chemistry, are yet to be fully understood. In addition, studies have pointed toward the instability of porous BN materials when exposed to humidity, which could significantly impact performance in industrial applications. Studies on porous BN performance and recyclability when employed in adsorption, gas storage, and catalysis remain limited, despite encouraging preliminary studies. Moreover, porous BN powder must be shaped into macrostructures (e.g., pellets) to be used commercially. However, common methods to shape porous materials into macrostructures often cause a reduction in the surface area and/or mechanical strength.

In recent years, research groups, including ours, have started addressing the challenges discussed above. Herein, we summarize our collective findings through a selection of key studies. First, we discuss the chemistry and structure of BN, clarifying confusion around terminology and discussing the hydrolytic instability of the material in relation to its structure and chemistry. We demonstrate a way to reduce the instability in water while still maintaining high specific surface area. We propose a mechanism for the formation of porous BN and discuss the effects of different synthesis parameters on the structure and chemistry of porous BN, therefore providing a way to tune its properties for selected applications. While the syntheses covered often lead to a powder product, we also present ways to shape porous BN powders into macrostructures while still maintaining high accessible surface area for interfacial processes. Finally, we evaluate porous BN performance for chemical separations, gas storage, and catalysis.

While the above highlights key advances in the field, further work is needed to allow deployment of porous BN. Specifically, we suggest evaluating its hydrolytic stability, refining the ways to shape the material into stable and reproducible macrostructures, establishing clear design rules to produce BN with specific chemistry and porosity, and, finally, providing standardized test procedures to evaluate porous BN catalytic and sorptive properties to facilitate comparison.

## 1 Introduction

Boron nitride (BN) is a well-known ceramic material. Its bulk cubic and hexagonal crystalline forms have been employed commercially for decades as abrasives, lubricants for paints and cosmetics, as well as components for high-temperature furnaces. Notably, we observe a renewed interest for this material in recent literature. We link this enthusiasm to new forms of the material that present distinct or additional properties to bulk cubic and hexagonal BN, namely: BN nanotubes, single-layer BN nanosheets (“white graphene”), and porous BN.

Here, we take a closer look at the latter form, whose porosity and chemistry make it relevant for interfacial processes. We present the latest findings around porous BN, as well as early and pioneering reports. We focus our discussion on the formation of porous BN, how this formation impacts its chemical and physical properties, and how these properties influence the performance of BN in interfacial applications including molecular separations, gas storage, and catalysis. This Account does not offer an exhaustive report of the literature on porous BN. Instead, we have selected studies that highlight our perspective on where the field is going and how it could progress further. As we observe a research growth, we also notice confusion in terminology, discrepancies in reports, and gaps in knowledge. Here, we highlight these challenges and propose ways to address them.

## 2 Structure and Chemistry of Boron Nitride

### 2.1 Structural Features

**Figure 1:**
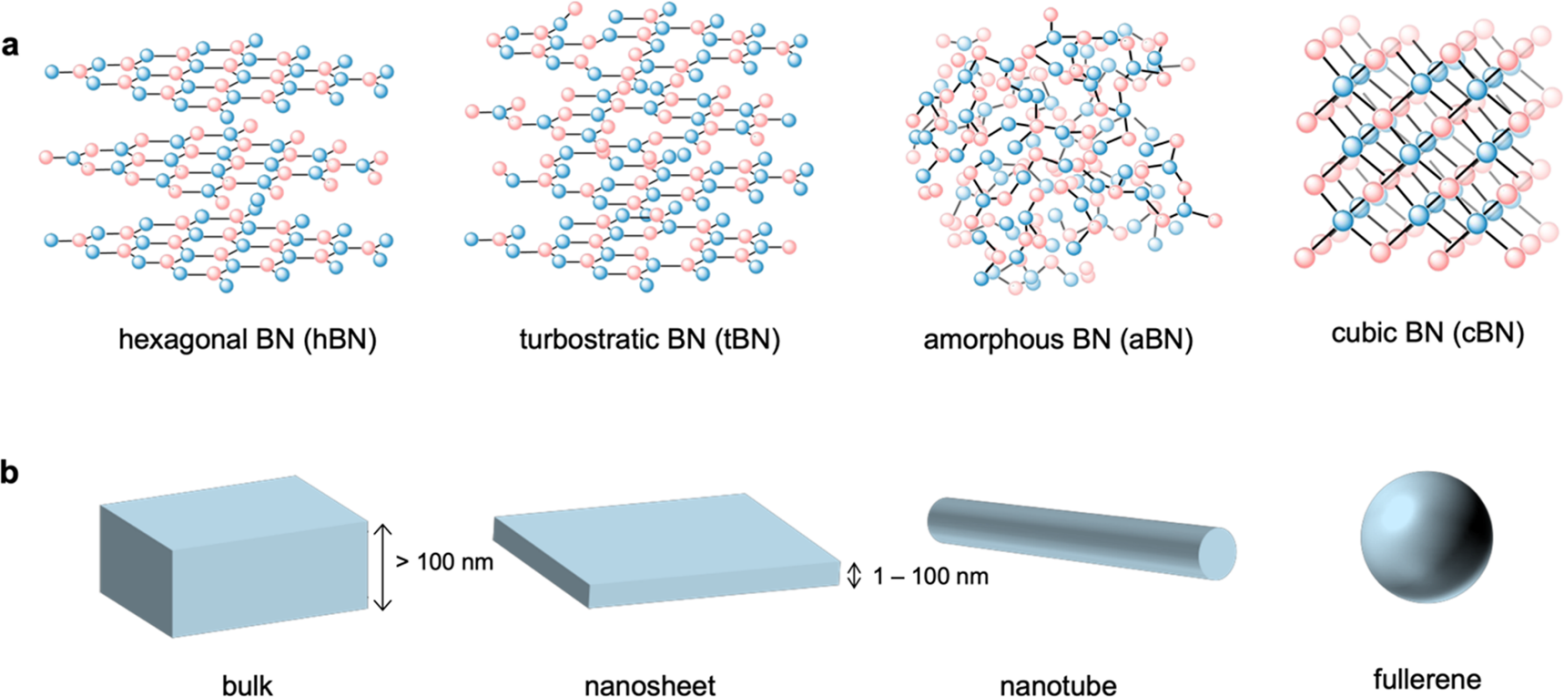
Overview of the forms of BN materials: (a) crystal and amorphous structures (not inclusive of all crystal structures); (b) forms in which one can produce BN materials.

BN is formed of an equal number of boron and nitrogen atoms that can arrange in crystalline, semicrystalline, and noncrystalline structures, with either sp^2^ or sp^3^ bonds. The main crystal structures of BN are hexagonal (hBN), rhombohedral (rBN), cubic (cBN), and wurtzite (wBN). hBN and rBN exhibit sp^2^ bonding, with different stacking arrangements (AA′ and ABC, respectively), while wBN and cBN are denser forms of BN with sp^3^ bonds. BN can also be turbostratic (tBN, described below) or amorphous (aBN) (Figure 1a). By increasing the temperature and pressure, BN can transition from tBN to hBN and finally to cBN.^1^

In addition, BN can exist as bulk, two-dimensional (2D) nanosheets, 1D nanotubes, and 0D fullerenes (Figure 1b). A nanosheet, according to ISO (ISO/TS 80004-11:2017), is a “nanoplate with extended lateral dimensions”, with the term nanosheet typically used to describe flakes of hBN. A nanoplate is defined as a “nano-object with one external dimension in the nanoscale and the other two external dimensions significantly larger” and is technically a more accurate term for flakes of hBN.

Confusion with the terminology around BN materials exists in the literature. For instance, terms like hBN, nanosheets, and turbostratic are often used interchangeably. Yet, tBN exhibits different properties from bulk hBN or few-layer hBN nanosheets, such as lower thermal and oxidative stability. hBN generally displays an AA′ stacking sequence, with each nitrogen atom in one layer eclipsing boron atoms in the upper (or lower) layer. tBN instead displays a “relative and random rotational angle or commensurate rotation between the layers” (ISO/TS 80004-13:2017). Following this, when characterized using X-ray diffraction (XRD), tBN shows only 001 peaks (002, 004, etc.) with three Miller indices and other peaks with two indices, typically 10 and 11. Few-layer hBN nanosheets or single-layer hBN can exhibit similar XRD patterns due to the reduced layer number. In powder form, individual few-layers of single-layer hBN particles stack with random orientations between the particles, leading to an XRD pattern like tBN. Microscopy techniques capable of atomic/nanoscale resolution, e.g., transmission electron microscopy (TEM) and atomic force microscopy (AFM), can help distinguish between few-layer hBN nanosheets and tBN.

Here, we focus on BN forms that exhibit high specific surface areas (>100 m^2^ g^–1^), excluding nanotubes and fullerenes. High surface area BN can be obtained via either (i) increasing the surface-to-volume ratio of the material or (ii) obtaining a microporous (pores <2 nm) and/or mesoporous (2–50 nm) BN material. Examples of high surface area BN materials produced via the first route include hBN nanosheets. These nanosheets are obtained through bulk hBN exfoliation using top-down methods. The highest theoretical surface area for hBN nanosheets is 2630 m^2^ g^–1^. When not in suspension, materials produced via this route display a lower specific surface area than the theoretical maximum, due to the restacking of individual particles. High surface area BN materials (up to 4800 m^2^ g^–1^ from theoretical predictions)^2^ derived from the second route are produced through bottom-up methods. They exhibit a crystallographic structure lacking stacking order, often identified as tBN or aBN, though some crystalline domains may be present. Here, we focus on materials obtained via the second route, which we refer to as “porous BN”. They generally exhibit surface areas well above 500 m^2^ g^–1^ and closer to 1000–1500 m^2^ g^–1^.

### 2.2 Chemical Features

**Figure 2:**
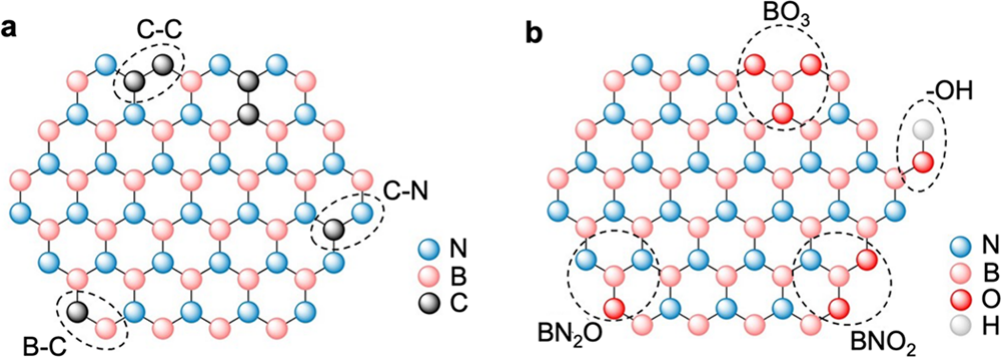
Schematic representation of (a) carbon sites in C-doped BN and (b) oxygen sites in O-doped BN.

Porous BN exhibits defects that take the form of lattice vacancies and/or carbon or oxygen atoms incorporated in the BN structure. These elements, also referred to as “impurities”, originate from the B-containing (for O atoms) and the N-containing (for C and O atoms) precursors employed to form BN. C atoms can replace B or N atoms, thereby forming C–B, C–N, and C–C bonds (Figure 2a).^3^ The C content ranges from a few at. % to 15–20 at. % depending on the synthesis conditions. The synthesis atmosphere, N_2_ or NH_3_, plays a role in the composition of BN, the latter favoring a lower C content.^4^ Higher C contents are possible, but we do not consider them here as the material would become BCN rather than BN. The removal of C atoms from the BN structure typically occurs between 400 and 900 °C depending on the atmosphere as carbon evolves in the form of CO_2_, HNCO, HCN, and CH_2_N_2_.^5,6^ On the other hand, O atoms preferentially replace N atoms, forming B–O, O–N, and O–H bonds within the basal planes and at the edges of the BN nanosheets (Figure 2b). Replacement of B atoms with O atoms causes structural deformation and is therefore not favored.^7^ O contents up to 20 at. % have been predicted, beyond which the BN structure becomes unstable.^8^ We recently reported the tuning of the O content via control of the synthesis parameters including the following: nature and ratio of the precursors, synthesis temperature, and atmosphere flow rate.^9^ We presented a response surface model to predict and experimentally tune the chemical composition of the resulting BN. Like for carbon, the O content tends to decrease upon increasing the synthesis temperature. Overall, defects confer porous BN with a “richer” chemistry than one could initially envision. O and C atoms allow the following: (i) further BN functionalization via grafting and (ii) modulation of its adsorptive properties, thereby opening the way for possible applications in separation, energy storage, and catalysis (Sections 5, 6, and 7). Yet, the presence of such impurities also causes porous BN to be susceptible to humidity as discussed in Section 2.3.

Beyond playing with the synthesis precursors, doping of porous BN using both nonmetals (i.e., C, O, P, F, S, Si) and metals (i.e., Pt, Pd, Ag, Au) can be used to dictate the chemistry of porous BN and to further tune BN’s properties. In Table 1, we include computational and experimental work related to bulk hBN as well as porous BN since the doping strategies employed for bulk hBN could transfer to porous BN. We note that BN precursors may also include metal (e.g., metal organic frameworks) and act as both BN precursor and dopant provider. Research in this area is still limited and deserves further exploration. Indeed, dopants help modulate the sorptive, magnetic, optoelectronic, and (photo)catalytic properties of BN, thereby expanding the fields of application. Despite the promise of doping BN materials, the number of studies on the topic remains limited, and we can only foresee a growth in this area. Examples of porous BN properties and their role in practical applications are presented in Sections 5–7.

**Table 1:** Studies Relating to the Doping of Porous BN^a^

dopant	BN structure	dopant content	dopant chemical environment	dopant role in the study	nature of the study
C	porous BN, hBN nanosheets (BNNS)	5–20 wt % C	basal planes	reduce bandgap, add sorption sites, add active catalytic sites	computational + experimental
O	porous BN, BNNS, hBN monolayer	5–20 at. % O	basal planes, edges	reduce bandgap, tune magnetic properties	computational + experimental
C, O	porous BN	8 at. % C,6 at. % O	basal planes	increase specific area and adsorption capacity	experimental
Si	BN nanotubes	0.08 at. % Si experimental, 5 at. % Si computational	basal planes	new synthesis technique, reduce bandgap	computational + experimental
P	porous BN, hBN monolayer	1–5 wt % P	basal planes	add sorption sites, reduce bandgap	computational + experimental
S	hBN, hBN monolayer		basal planes	reduce bandgap, reduce electrical resistivity	computational + experimental
F	hBN monolayer		basal planes	reduce bandgap	computational
F, C, O	porous BN		basal planes	reduce bandgap, add sorption and catalytic sites	computational
Cl	hBN monolayer		basal planes	reduce bandgap	computational
metals (Sc, Ti, V, Cr, Mn, Fe, Co, Ni, Cu, Zn, Mo, Ru, Rh, Pt, Pd, Au, Ag)	porous BN, hBN monolayer, BN nanobelts		composite form within and on monolayer surface basal planes	introduce plasmonic heating, reduce reaction-limiting potential, reduce bandgap, tune magnetic properties, create electric field within material	computational + experimental

a Full details in the Supporting Information, Table S1.

### 2.3 Water Stability

Considering the unavoidable exposure to water/moisture in most applications, one must assess the material’s water stability. Early experiments pointed toward the hydrolytic instability of porous BN, which decomposes into ammonia and boron trioxide as per reaction 1.^10,11^ More recently, studies by Florent and Bandosz and our group observed a dramatic decrease in surface area upon exposure to moisture or liquid water.^3,12^
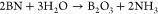
1

Both groups have shown that, while hBN is known to be hydrophobic, C and O atoms weaken the stability of porous BN toward water. The B–C bond appeared particularly unstable, and O atoms limited the hydrophobicity of porous BN. Therefore, reducing the C and O contents can help enhance the stability of porous BN. This can be achieved by raising the synthesis temperature of porous BN (from ∼1000 to 1500 °C), which leads to further removal of C and O atoms in gaseous forms and enhances the phase purity and crystallinity. As a result, the material obtained at 1500 °C exhibited better stability, likely due to higher crystallinity and fewer defects in the structure. Yet, a trade-off exists between stability and surface area/porosity (which decreases at 1500 °C) and must be considered depending on the targeted application.

## 3 Formation of Porous BN Powder

Here, we discuss the general requirements of porous BN synthesis, the current knowledge around its formation mechanism, and the synthesis parameters known to influence the chemical and structural features of the resulting material. As explained in Section 2.1, we focus the discussion on bottom-up synthesis routes that form porous BN and exclude top-down routes. Among the bottom-up routes, chemical blowing relies on blowing H_2_ gas through a previously heated B-containing and N-containing precursor, creating B–N–H polymer structures with large bubbles, which then collapse to form hBN nanosheets.^13^ Porous BN can also form via chemical vapor deposition, but the resulting material does not exhibit a high surface area. In solvothermal synthesis, N- and B-containing precursors are heated in an autoclave, usually <500 °C.^14^ The method is scalable but requires a solvent and therefore a separation step to remove it. Template-based synthesis employs a porous template, and BN is grown on its surface prior to template removal.^15^ Another bottom-up synthesis route is the direct high-temperature synthesis under a controlled atmosphere. This route is practical in terms of yield and simplicity, leads to high surface area, and allows the producer to tune the resulting product features. We limit the discussion below to this direct high-temperature synthesis route.

### 3.1 Overview

**Figure 3:**
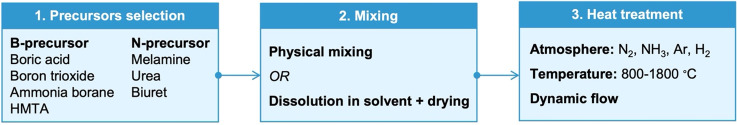
Schematic of the synthesis steps to produce porous BN with examples of precursors (HMTA = hexamethylenetetramine).

Figure 3 illustrates the bottom-up synthesis route steps. In short, B- and N-containing precursors, either solid or gaseous, are subjected to a high-temperature treatment (>800 °C) under a dynamic flow of a controlled atmosphere. Possible gases for the synthesis/reaction atmosphere include N_2_ and NH_3_, which can also act as N-containing precursors. The solid B- and N-containing precursors can be first dissolved in a solvent and dried to form a solid intermediate that is then heated. Yet, we demonstrated that physical mixing of the precursors often suffices to generate porous BN.^5^ Further, we showed that using more than one N-containing precursor exhibiting different thermal decomposition profiles leads to enhanced surface area and enables finer tuning of the porosity.^16^ We attributed this to the wider temperature range for precursors’ decomposition, causing a release of gases/porogens over a larger temperature range and creating a more complex pore structure. Typically, surface areas would range from 400 to 1500 m^2^ g^–1^, but this would vary depending on the synthesis method used.

### 3.2 Mechanism

**Figure 4:**
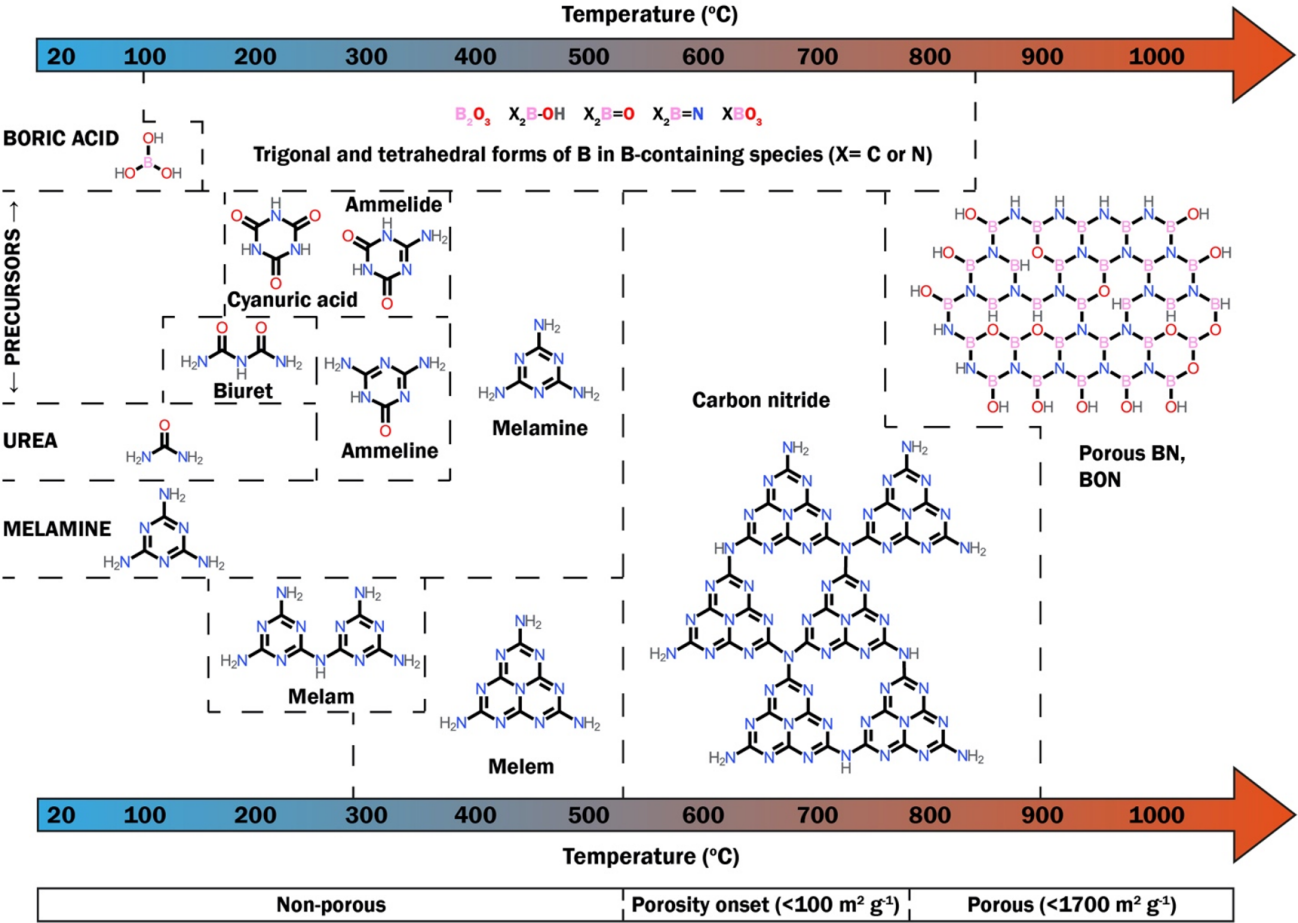
Proposed species evolution during the formation of porous BN from boric acid, melamine, and urea under N_2_ atmosphere up to 1050 °C. Reproduced with permission from ref (6). Copyright 2021 American Chemical Society.

To design the synthesis process of porous BN and make informed decisions on the parameters (e.g., temperature, flow rate, reaction atmosphere, precursors), one must understand the material’s formation mechanism. Studies on porous BN formation have investigated the reaction of ammonia with a boron precursor^4,17,18^ or the use of a carbon nitride intermediate prior to conversion into BN.^19^ These studies have generated useful knowledge, yet they do not allow a universal and generalizable understanding of the overall porous BN formation mechanism. Using boric acid, melamine, and urea as precursors and a heat treatment at 1050 °C under N_2_, we tried to address this gap in a detailed study.^6^ We investigated the nature of the intermediates formed at regular temperature intervals up to 1050 °C using spectroscopic and analytical tools. From these analyses, we highlighted that the two N-containing precursors, melamine and urea, act as both precursors and porogens. Up to about 600 °C, the three precursors degrade and evolve into subproducts, such as biuret, cyanuric acid, and ammelide. We also demonstrated that porous BN formation goes through the formation of carbon nitride from about 600 °C, which then reacts with B-containing species such as B_2_O_3_, X_2_B–OH, X_2_B=O, and X_2_B=N (where X = C or N) to form BN from 700 °C. A schematic overview of the mechanism is presented in Figure 4. While this mechanism relates to a set of chemical precursors, the similar chemistry and thermal decomposition pattern of typical N-containing precursors for porous BN suggest that this route might be generalizable.

### 3.3 Effects of Synthesis Parameters

We briefly discuss below the effect of synthesis parameters on porous BN and provide a summary in Table 2.

- **The nature and relative quantity of the precursors** help tune the porosity of porous BN^16^ and the C and O contents.^9^ Furthermore, using several N-containing precursors with varied thermal decomposition profiles enhances the surface area and porosity of the final material.^6,16^ Finally, primary and secondary amine precursors are preferred over tertiary amine precursors as they lead to a lower energy barrier for reaction with boric acid.^8^
- **The nature of the reaction atmosphere** influences both the porosity and composition. Synthesis under NH_3_ as opposed to N_2_ provides more reactive N atoms, ensures the complete reaction of the B-containing precursors, and limits carbonization, thereby enhancing the porosity and limiting the presence of carbon impurities, respectively.^4,15,20^
- A minimum of ∼800 °C is required to obtain porous BN, and higher temperatures contribute to getting “purer” (i.e., fewer O atoms) and enhanced porosity.^6^ Beyond a certain temperature, though, crystallization occurs, causing the surface area to decrease.^4,9^ The exact temperature at which this transition happens remains unknown.
- The **flow rate** drives the reaction gas residence time and that of the gaseous intermediates formed. Hence, it can a priori impact how these species react/interact with the solid precursors and reaction intermediates and thereby the composition of the final porous BN. However, we have not observed a strong correlation between flow rate and BN composition, though in some instances, a higher flow rate caused fewer C and O impurities.^9^

**Table 2:** Synthesis Parameters and Their Impact on the Resulting BN Material Based on the Findings from the Studies Referenced in the Table

variable	impact	comments	ref
B-precursor	purity	O content ↑: purity ↓	(16)
N-precursor	reaction rate	primary or secondary amines: reaction rate ↑	(9)
	purity	primary or secondary amines: purity ↑	(9)
	porosity/surface area	>1 N-precursor: porosity and SSA ↑	(6), (16)
mixing method	porosity/surface area	drying time ↑: porosity and SSA ↑	(5)
		physical mixing: high porosity	(5)
temperature	purity	*T* ↑: O and C contents ↓	(6)
	porosity/surface area	800–1000 °C: porosity and SSA ↑	(6)
		1000–1500 °C: crystallization ↑ and porosity and SSA ↓	(4), (9)
atmosphere	purity	NH_3_ vs N_2_: carbonization ↓ and purity ↑	(4, 15, 20)
	porosity/surface area	NH_3_ vs N_2_: porosity ↑	(4, 15, 20)
flow rate	purity	flow rate ↑: O and C contents ↓	(9)

## 4 Shaping of Porous BN

Powders (discussed in Section 3) are rarely a usable form for industrial applications: they are difficult to handle and often suffer from poor mass transfer, recyclability, and mechanical strength. One must shape porous materials into robust structures and maintain the desired features of the initial powder. Here, we discuss the types of shaped porous BN and how they are produced.

### 4.1 Types of Shaped Porous BN

Shaped porous BN can be classified into aerogels, structured BN, and pellets. These names are not always consistently or accurately employed, and we take the opportunity to review their definition. Following the International Union of Pure and Applied Chemistry (IUPAC) terminology,^21^ an aerogel is a “gel comprised of a microporous solid in which the dispersed phase is a gas”. The ISO definition (ISO 22482:2021, 3.1.1) refers to an “insulating material that has high porosity derived from a nanoporous structure formed by replacement of the liquid component of a gel with air”. An aerogel forms an open-cell solid foam composed of a network of interconnected nanostructures. The terms aerogel and foam are often used interchangeably. Here, for clarity, we solely refer to the IUPAC term aerogel. The term “structured BN” (no IUPAC definition) refers to a macroscale form of BN that is denser than an aerogel but not produced by compaction of powder and not necessarily demonstrating a reproducible and controlled geometric macroshape. Finally, the term “pellet”, which does not have a formal IUPAC definition either, refers to an agglomerate of solid particles formed using varied processing methods, including compression. Overall, the density of the material typically increases from aerogel to structured BN to pellet. Both aerogels and pellets are monoliths, which, following IUPAC nomenclature,^21^ are a “shaped, fabricated, intractable article with a homogeneous microstructure which does not exhibit any structural components distinguishable by optical microscopy”.

### 4.2 Production and Properties of Shaped Porous BN

**Figure 5:**
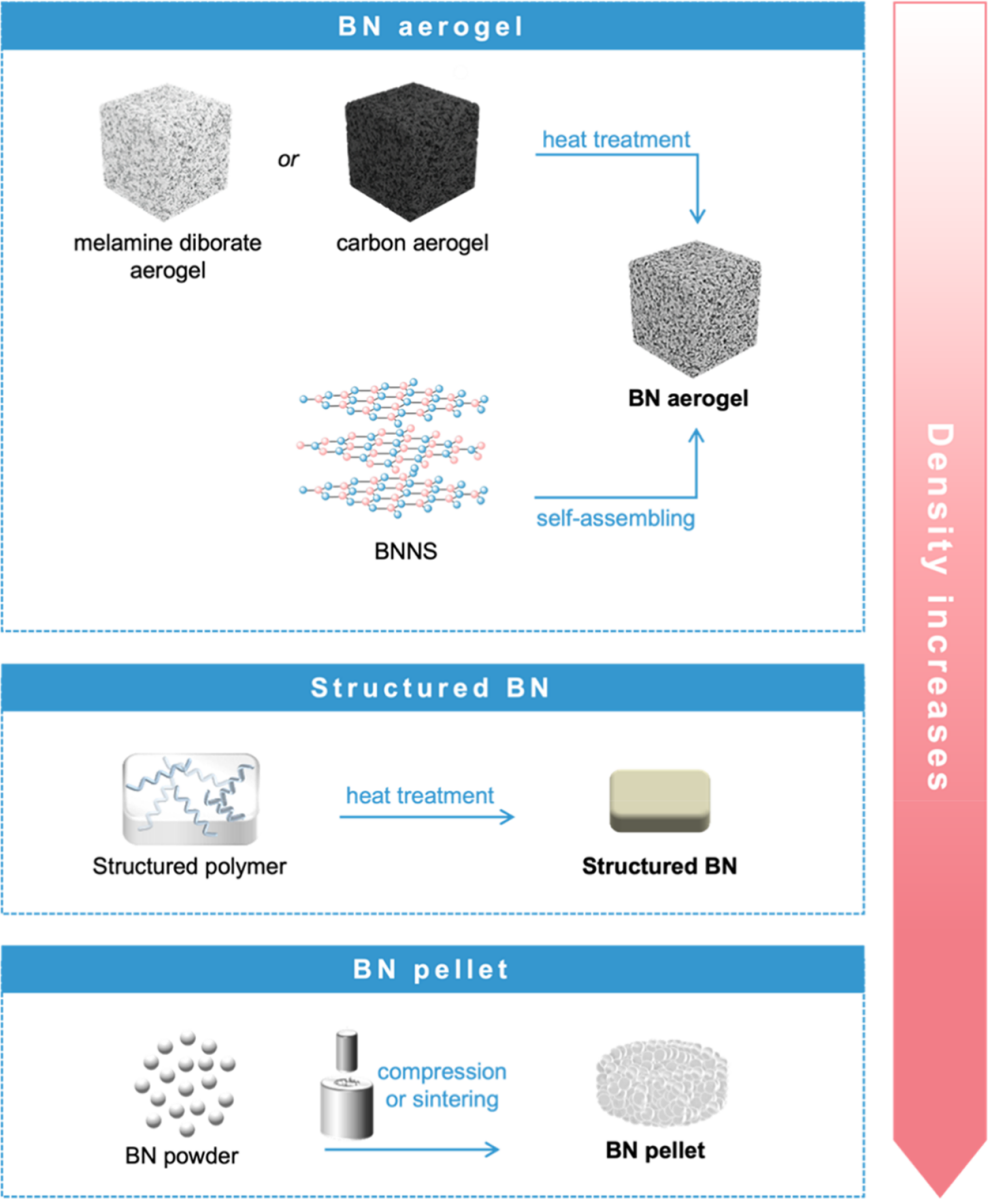
Schematic representation of the types of shaped BN and their synthesis routes.

Methods to produce shaped porous BN include the following: (i) templated-assisted methods which produce a shaped porous BN without prior formation of a porous BN powder and (ii) powder-processing methods which use an already formed porous BN powder. The formation of aerogels most often falls into the former category, that of pellets belongs to the latter category, while that of structured BN may belong to either depending on the synthesis process (Figure 5).

#### 4.2.1 Template-Assisted Shaping Methods

##### Aerogels

BN aerogels are obtained following one of three methods (Table S2), namely: (i) carbon aerogel-based method, (ii) polymer-based method, and (iii) BNNS-based method. All methods rely on forming a shaped intermediate usually obtained via freeze-drying followed by heat treatment under NH_3_, N_2_, or Ar, but they differ by the nature of the intermediate. In the carbon aerogel-based method, the shaped intermediate is a carbon aerogel.^22−24^ In the polymer-assisted method, a polymeric aerogel rich in B and N atoms, often a melamine diborate aerogel, acts as the intermediate aerogel.^24,25^ Yet, Cao et al.^26^ found that synthesizing a melamine diborate powder rather than a melamine diborate aerogel may suffice to form porous BN. Finally, the BNNS-based method relies on the formation of a BNNS hydrogel intermediate. The BNNS are self-assembled into a hydrogel via cross-linking and/or physical interactions. The hydrogel is then freeze-dried to obtain the BNNS aerogel.^27^ This method could arguably be categorized as bottom-up as it relies on hBN powder formation as an initial step.

BN aerogels exhibit low density, thermal conductivity, and dielectric constant, high surface area, and good mechanical flexibility. BN aerogels exhibit low bulk densities (i.e., weight of sample per unit volume) between 0.1 and 100 mg cm^–3^ (Table S2), which benefits applications requiring high gravimetric performance and light materials. Mechanical strength is another important property for applications relying on porous materials. Indeed, materials are always exposed to mechanical stress such as operational vibration and compression, e.g., weight of the packed adsorbent. Owing to its interparticle connections, BN aerogels present good elasticity, suggesting a high mechanical strength under compression.^22,25−27^

##### Structured BN

Structured BN displays high bulk density and improved mechanical stability compared to BN powder. Unlike aerogels, studies on such materials remain rare, due to the difficulty of maintaining high surface area and high density concurrently. Yet, such a combination of properties is needed in applications requiring high bulk density and volumetric performance, e.g., gas separation or gas storage. Recently, our group synthesized a structured BN by employing mechanically stable porous melamine–formaldehyde resin as a precursor.^4^ By optimizing the pore density of the resin, we synthesized a porous (Brunauer, Emmett, and Teller (BET) specific surface area (SSA): (1575 ± 92) m^2^ g^–1^), mechanically robust shaped BN (hardness: (66 ± 5) MPa) with a relatively high bulk density (0.31 g cm^–3^). The high bulk density and surface area led to a high volumetric surface area (473 m^2^ cm^–3^). Although the shape of the precursor and thus that of BN could not be controlled, a possible way forward would be to control the curing speed of the resin in a mold.

#### 4.2.2 Powder-Processing Methods

These methods consist of agglomerating (and partially aggregating) particles into a shaped structure. This agglomeration can happen via sintering or via mechanical compaction. We focus first on a sintering route. Taking advantage of the ceramic nature of BN, Bernard and Miele^28^ sintered porous BN powder using a spark plasma sintering method. The rapid heating rates suppressed the grain growth and led to a controlled microstructure and densification process. The sintered structured BN presents a good BET SSA, albeit lower than that of the powder precursor (430 vs 1100 m^2^ g^–1^), and a high bulk density (0.786 g cm^–3^).

Mechanical compaction usually requires adding inorganic and/or organic binders to favor cohesion between the porous material particles. The compression and the presence of an inactive component in the pellet decrease the adsorption/catalytic performance of the material compared to its powder counterpart, due to pore collapse, pore blockage, or reduced porosity. Yet, such a method is very common to produce zeolite pellets and is investigated for other porous materials such as metal organic frameworks (MOFs). A study has investigated the formation of porous BN pellets via compression without the addition of binders.^16^ While the resulting pellet maintained a good porosity, its mechanical strength and bulk density were not investigated. We encourage further studies in this area to fill in the knowledge gaps.

## 5 Porous BN for Molecular Separations

### 5.1 Separations Studied

Porous BN materials exhibit high specific surface areas, tunable pore structures, a bond polarity (basic N atoms and Lewis acidic B atoms), and high thermal stability, making them attractive adsorbent candidates. Table 3 summarizes studies in which porous BN materials were employed for liquid/vapor- and gas-phase separations. Note: comparing the adsorption performance of porous BN from different studies is challenging as materials are tested under different conditions. For example, in water-cleaning applications, the starting concentration of pollutants (*C*_0_ in Table 3) varies from a few mg L^–1^ to over 1 g L^–1^; the concentration of porous BN, the pH of the solution, and the experiment time also vary greatly.^29^

**Table 3:** Separation Studies Using Porous BN as Adsorbent, Showing the Type of Separations (Split into Liquid/Vapor and Gas Phase), Example of Species Involved, Adsorption Conditions (Initial Concentration of Pollutant (*C*_0_)), Quantity Adsorbed (Maximum Adsorbed Amount (*q*_max_)), and Sorption Mechanisms Suggested^a^

separation in liquid/vapor phase	example species	*C*_0_ range studied	*q*_max_ range	sorption mechanisms suggested
		(mg L^–1^)	(mg g^–1^)	
trace metal removal from water	Cu^2+^	200–1870	200–819	mainly ion-exchange interactions dominated by electrostatic and surface complexation; hydrogen bonding and physisorption also reported; increased adsorption in the presence of defects and higher SSA
	Pb^2+^	6–200	204–845	
	Cr^3+^	52–100	120–387	
	Ce^3+^	52	282	
	Ni^2+^	52–700	95–235	
	Co^2+^	52	215	
	Cd^2+^	200–600	107- 561	
	As^5+^	1–50	5–32	
removal of dyes from water	Methylene Blue	10–350	107–13973	mainly π–π stacking interactions; electrostatic interactions, physisorption
	Methyl Orange	40	298–395	
	Rhodamine B	3–100	76–992	
	Basic Yellow	90	424	
	Congo Red	50–130	307–782	
PFAS from water	PFOS	200	∼57	electrostatic interactions
	PFDA	50	∼90	
oils and organic solvents	engine oil		18000–56800	capillarity effect, pore filling, SSA
	ethanol		14000–25000	
	acetone		25000	
	toluene		16000–32000	
	toluene/*n*-heptane/methanol		∼600	
	cyclohexane		5000	
removal of biowaste from water	tetracyclines	20–300	118–1100	π–π stacking interactions, electrostatic interactions, van der Waals forces
	levofloxacin	20–300	318	
	sulfamethazine	20–300	19	
desulfurization of oil	dibenzothiophene (DBT) in *n*-octane	500–800	35–65 mg S g^–1^	Lewis acid–base interactions
iodine removal	iodine vapor		2120	physisorption, Lewis acid–base interactions
	iodine/*n*-hexane	50	61	

separation in gas phase	species involved	*T* (°C)	*P* (bar)	*q*_max_ range (mmol g^–1^)	sorption mechanisms suggested
precombustion carbon capture	CO_2_/H_2_	25	20	CO_2_: ∼8	physisorption; pore characteristics and electrostatic interactions key at low pressure; at high pressure volume and SSA play a key role
		25	40	CO_2_: ∼19	
postcombustion carbon capture	CO_2_/N_2_	25	1	CO_2_: 1.6–4.6	
				N_2_: 0.2–0.3	
natural gas sweetening	CO_2_/CH_4_	25	1	CO_2_: 1.6–3.9	physisorption; electrostatic interactions affect less CH_4_
		25	70	CH_4_: 0.6–0.7	
				CH_4_: ∼9.0	
formaldehyde from air	HCHO	25	1	0.63	physisorption, hydrogen bonding, π–π conjugation
				(20 ppm of C_0_)	
light hydrocarbons separation	N_2_, CH_4_, C_2_H_4_, C_2_H_6_, C_3_H_6_, C_3_H_8_	25	1	N_2_: 0.1	pure physisorption (dispersion forces)
				CH_4_: 0.5	
				C_2_H_4_: 2.1, C_2_H_6_: 3.0	
				C_3_H_6_: 4.5	
				C_3_H_8_: 5.5	
ammonia	NH_3_	25	1	NH_3_: 5.3, CO_2_: 0.9	physisorption, Lewis acid–base interactions, and H-bonding

a Full details in the Supporting Information, Table S3.

As seen in Table 3, the removals of organic and inorganic pollutants from water are the most widely studied separations.^29,30^ Among the range of contaminants tested are common pollutants such as dyes, metals, biowaste, perfluoroalkyl substances (PFAS), oils, and organic solvents. The performance of porous BN in water-cleaning applications is often comparable or superior to that of other adsorbents such as activated carbons, which may explain the recent explosion of studies in this area.^29,30^ Of particular promise is the removal of oils and organic solvents from water, in which porous BN showed good performance and recyclability.^24,31,32^ Often BN materials employed in oil adsorption studies are microporous aerogels with low surface area.^31^ Multiple studies demonstrated BN’s potential in the desulfurization of oil.^33^ Less investigated yet promising liquid-phase separations include iodine adsorption.^34^

In gas-phase separations such as carbon capture and natural gas sweetening, the performance of porous BN materials is generally lower than that of state-of-the art adsorbents such as MOFs and activated carbons, likely due to the presence of fewer active sites and/or lower microporosity.^35^ Approaches such as C-doping can improve CO_2_ adsorption by increasing the density of micropores and defects.^16,35^ High levels of C-doping could, however, affect some of the other properties, such as thermal and oxidative stabilities. Other less studied gas-phase separations include the adsorption of ammonia, the adsorption of formaldehyde, and the separation of light hydrocarbons.^36−38^

### 5.2 Separation Mechanisms and Influencing Factors

#### 5.2.1 Textural Properties

As adsorption is a surface-based phenomenon, high SSAs are generally key to improving the adsorption capacity. In porous BN materials, we previously showed that for similar surface chemistries, the adsorption amount of organic vapors and CO_2_ increased with SSAs.^16,32^ Pore size and volume also matter. For example, in CO_2_ adsorption studies, pore size is critical, with micropores and ultramicropores (distance between opposite walls of slit/pore <0.7 nm) known to be desirable pore sizes. However, pore volume becomes key at high pressures as molecules occupy larger pores.^35^

#### 5.2.2 Defects

Structural defects can become active adsorption sites as they can increase the SSA and pore volume of BN materials. They can also expose more reactive edges in which functional groups are likely to bind. Chemical defects such as inclusions of heteroatoms can alter the chemistry of porous BN, and their role in adsorption cannot be easily isolated from the role of functionalization/doping and structural properties. For example, CO_2_ sorption performance improves in more defective BN, which exhibits higher micropore volume and carbon and oxygen contents than pristine BN.^35^

#### 5.2.3 Electrostatic Interactions

Because of the local polarity of B–N bonds and some of the adsorbate molecules, electrostatic interactions often play a major role. For instance, the removal of metal ions from water is affected by the solution pH which influences the surface charge of BN adsorbents, thereby affecting the electrostatic interactions between adsorbent and adsorbate.^30^

#### 5.2.4 π–π Stacking Interactions

Delocalized electrons from sp^2^-hybridized BN can interact with electrons in π bonds of adsorbates. This adsorption mechanism is key in the removal of organic pollutants with aromatic rings.^30^ Adsorption is influenced by the number and location of aromatic rings in organic pollutants, which is attributed to differences in the π electron structure of adsorbates, affecting π–π stacking interactions.^39^

#### 5.2.5 H-Bonding

H-bonding between formaldehyde (HCHO) and hydroxyl or amine groups in functionalized porous BN confers the material its high performance in the adsorption of HCHO.^38^ H-bonds were also identified as a minor contributor to NH_3_ and CO_2_ adsorption, through the −OH and −NH_2_ groups present at the edges of porous BN.^37^

#### 5.2.6 Surface Chemistry

Functional groups or impurity atoms present in porous BN can affect the chemical interactions described above, by either enhancing or reducing BN’s adsorption capacity. The presence of impurities (e.g., C or O atoms) can also increase the number of defects and affect structural properties. For example, carbon doping was shown to improve CO_2_ sorption capacity due to an increase of defects and higher electrostatic interactions.^35^ The presence of −OH functional groups was shown to increase the adsorption of formaldehyde due to H-bonding.^40^

## 6 Porous BN for Gas Storage

The high porosity of porous BN makes it attractive for gas storage. Typically, gases (e.g., methane, hydrogen) are stored as liquified gas under cryogenic conditions or as compressed gas under extremely high pressure. Storage in a porous adsorbent can lead to milder storage conditions, providing possible safety and cost advantages. We present below studies that investigated H_2_ and CH_4_ storage in porous BN. We note here again that this Account focuses only on BN samples that exhibit high specific surface areas (i.e., >100 m^2^ g^–1^).

### 6.1 H_2_ Storage

Table 4 summarizes studies reported on H_2_ storage in porous BN. Note: comparison of maximum adsorption capacity of different BN materials is challenging, as the temperature, pressure, and type of adsorption capacity reported (e.g., net, excess, or absolute) might vary between studies. Here, we compare the effects of surface areas and dopants on H_2_ adsorption under the same testing conditions. These studies highlight that H_2_ storage in porous BN occurs via a combination of physi- and chemisorption or pure physisorption. These conclusions contradict the pioneering work of Wang et al.^41^ who presented a pure chemisorption mechanism. The studies also collectively point to the importance of three factors for H_2_ adsorption on porous BN: surface area, pore size, and surface chemistry.

**Table 4:** Gas Storage Studies Using Porous BN as Adsorbent, Showing the Specific Surface Area of the Material and the Performance Metrics at Given Conditions^a^

gas stored	SSA_BET_	*T*	*P*	gas uptake	desorption level
	(m^2^ g^–1^)	(°C)^b^	(MPa)	(wt %)	(%)^c^
H_2_	150	RT	10	1.8	30
	210	RT	10	2.6	30
	260	RT	10	2.9	20
	790	RT	10	4.2	50
	1560	–196	0.1	1.07	N/A
	215	25	10	4.07	N/A
	1150	–196	1	2.3	100
	1690	25	3	5.6	84
	1040–1070	–196	0.1	1.41–1.6	N/A
			1	1.9–2.14	N/A
	1900	–196	1	2.6	100
	540	RT	5	5.7	89
	400–1290	–196	0.1	0.98–1.35	N/A
			1	1.65–2.33	100
CH_4_	440–720	0	0.1	0–3	N/A
	1050	0	0.1	1.1	N/A
	1500	25	7	13.6	N/A

a Full details in the Supporting Information, Table S4.

b RT stands for room temperature.

c Percentage of gas released when the pressure is reduced to ambient conditions.

High surface area favors H_2_ storage by providing more active sites and surface for interactions between BN and H_2_. For instance, porous BN has been shown to adsorb 2.3 wt % H_2_ at 1 MPa, −196 °C, while hBN adsorbed 0.1 wt % under the same conditions.^42^ Other studies have confirmed that storage capacity is related to BN’s surface area.^43^ H_2_ storage capacity can also be influenced by the adsorbent’s pore size distribution. Using samples with similar surface area and varied pore sizes, Weng et al.^44^ demonstrated that ultranarrow pores (0.4 and 0.6 nm) limited H_2_ storage at high pressure compared to larger pores (1.1 nm). Using first-principles calculations, Dai et al.^2^ found that H_2_ adsorption is energetically unfavorable in BN with pore sizes smaller than 0.44 nm, while larger pore sizes eliminate the effect of the repulsion between BN and H_2_.

The presence of elements other than B and N in porous BN modifies the electronic and chemical properties of BN and therefore its H_2_ adsorption properties. Studies have reported enhanced H_2_ storage capacity via O-doping^45^ and C/O-codoping.^46^ Metallic dopants have also been considered to enhance H_2_ storage for hBN, which is beyond the scope of this Account.^47^

### 6.2 CH_4_ Storage

Compared to H_2_ storage, CH_4_ storage using porous BN has been less studied. To gain insight, we include here studies that investigated BN forms other than porous BN. Using density functional theory calculations, Seyed-Talebi and Neek-Amal^48^ found CH_4_/hBN exhibited a shorter equilibrium distance than CH_4_/graphene, indicating preferential sorption on hBN. The authors observed little charge transfer from methane to hBN, revealing a chemisorption process. Ganji et al.^49^ found a different result with purely physical interactions between CH_4_ and BN nanosheets. A few experimental studies have been reported on CH_4_ storage in porous BN, including one from our group.^4,50,51^ The different testing conditions make a direct comparison impossible. In our work, we reported the gravimetric and volumetric CH_4_ adsorption capacity. The latter is key for real-world applications where the available space for fuel is limited. The tested BN powder and shaped BN exhibited volumetric CH_4_ capacities of 42 and 59 cm^3^ cm^–3^ (at 25 °C, 0.1 MPa), respectively. The latter value surpassed that of conventional porous materials (e.g., MCM-41) and emerging ones (e.g., porous organic cage). Overall, the insights provided by these studies, albeit encouraging, remain limited, which prevents a comprehensive understanding of the CH_4_ adsorption mechanisms in porous BN and the role of BN’s chemical and structural features.

## 7 Porous BN for Catalysis

### 7.1 Heterogeneous Catalysis

The reactive sites of porous BN owing to the presence of O and C atoms and its surface area make it a potential candidate for catalytic reactions. The available surface area favors the initial adsorption step while the chemistry of BN can be used to modulate the adsorption and desorption of reactants and products, respectively, and it can also influence the reaction activation process. Grant et al. showed that even hBN contains O atoms and can act as a heterogeneous catalyst.^52^ Since then, other studies have reported using porous BN or hBN as an intrinsic heterogeneous catalyst or a catalyst support (Table 5).^53,54^ The dominant factor driving catalytic activity is the presence of O-containing groups and more specifically B–OH and B–O–O–B edge groups.^55^ For aerobic oxidative desulfurization, zinc (Zn) salt has been used as a template to develop porous BN, and the active site for the oxygen activation is composed of Zn nanoparticles and N-terminated edges.^56^

**Table 5:** Summary of Catalysis Studies Using Porous BN as a Catalyst, Showing the Type of Catalysis, the Targeted Reaction, and Some of the Key Performance Indicators^a^

Heterogeneous catalysis		
reaction studied	reaction conditions	reactant mixture
oxidative dehydrogenation of alkanes	460–490 °C	multiple ratios
		alkane:O_2_:N_2_
methane oxidation	690–720 °C	2:1:4
		methane:O_2_:N_2_
styrene epoxidation	80 °C	26.5 mmol of *t*-butyl hydroperoxide
		25 mL of acetonitrile
		10 mmol of styrene
aerobic oxidative desulfurization	150 °C	40 mL of oil (500 ppm of dibenzothiophene)
acetylene hydrochlorination	200–280 °C	1:1–1:2
		HCl:acetylene

Photocatalysis					
reaction	reaction phase	irradiation range	light source	sacrificial agent	production rate
H_2_ generation	liquid	200–2500 nm	300 W Xe lamp	triethanolamine	3.6–47.1 μmol H_2_g^–1^ h^–1^
CO_2_ photoreduction	gas	>300 nm	300 W Xe lamp	H_2_O	0.2–12.5 μmol CO g^–1^ h^–1^
				H_2_	1.5 μmol H_2_ h^–1^
					3.3 μmol H_2_g^–1^ h^–1^

Electrocatalysis					
reaction	catalyst	formation rate	solution	faradaic efficiency/electron transfer number	time/durability
N_2_ fixation to NH_3_	h-BNNS	18.2–22.4 μg NH_3_ h^–1^ mg_cat_^–1^	0.1 M HCl	4.7–5.5%@–0.70–0.75 V vs relative hydrogen electrode	2 h
	porous BN		0.1 M Na_2_SO_4_		
oxygen reduction reaction	Pt/porous BN	<0.6% H_2_O_2_	0.1 M HClO_4_	>3.99 electrons	10000 cycles for specific conditions
	BN nanotubes		0.5 M H_2_SO_2_		
	BNNS				
	sputter-deposited BN				

a Full details in the Supporting Information, Table S5.

### 7.2 Photocatalysis and (Photo)electrocatalysis

While hBN is an insulator, we and others have shown that the presence of defects in hBN and porous BN can reduce the bandgap and allow light absorption in the UV and visible regions.^8,9,57^ So far, porous BN has been employed as a photocatalyst for water splitting^58^ and carbon dioxide photoreduction,^57^ exhibiting semiconducting behavior and appropriate redox potentials and activity under UV–vis and sometimes visible light (Table 5). For CO_2_ photoreduction, the presence of paramagnetic OB_3_ centers seems to play a role in the photoactivity.^57^ Work on BN/semiconductor heterojunctions for photocatalysis has also been reported.^59^ These studies focus on using the heterojunctions for water pollutant photodegradation or H_2_ generation, during which BN promotes charge separation. So far, studies point to the fact that BN can act as a “pure” photocatalyst or as a cocatalyst. In the former case, BN behaves as a semiconductor where charge separation occurs followed by the reduction of activated molecules. In the latter case, BN serves as an acceptor of photoexcited holes or electrons, thereby reducing charge recombination and enhancing photocatalytic activity. While the work highlighted here provides a proof-of-concept to use porous BN as a photocatalyst, many unknowns remain, e.g., the role of the chemical, structural, and optoelectronic features of porous BN on the photocatalytic performance and reaction mechanisms.

Porous BN is also considered as a possible (photo)electrocatalyst, for oxygen reduction reaction (ORR) and N_2_ fixation into NH_3_ (Table 5).^60,61^ For ORR, a common conclusion is that BN acts as an electrocatalyst, exhibits good stability, and reduces the overpotential compared to commercial C-containing electrocatalysts. Studies show that doping with other elements can improve peak potential and current density and favor electron transfer. For N_2_ fixation, the activity of BN is related to its porosity, which favors exposure to active catalytic sites^61^ as well as the presence of unsaturated boron at the edges, which activates N_2_ molecules.^60^ We note that these reactions occur in the aqueous phase, which may challenge the prolonged usage of porous BN.

## 8 Conclusions and Perspectives

We presented selected studies on porous BN to highlight the state of knowledge on the material synthesis, its chemical and structural features, as well as its applications in interfacial processes. This collection reveals increasing research on this material, particularly for molecular separation, gas storage, and catalysis. Porous BN appears as a promising material for these applications owing to its chemical structure, high specific surface area and thermal stability, and tunability. Its chemical, sorptive, optoelectronic, and magnetic properties can be altered by the creation of defects and/or inclusion of functional groups and heteroatoms/dopants in its structure. While enthusiasm for porous BN grows, our analysis also leads us to propose cautionary notes and suggestions.

### Following an Accurate Terminology

The names used for powder and shaped porous BN are not always consistent or accurate. Here, we have tried to review the definition of each BN form, and, when possible, we have used the IUPAC and ISO naming conventions. We encourage researchers to employ a range of techniques to characterize the form/forms of BN they work with and select the most accurate names. Note: a given sample may contain different forms of BN (e.g., a porous BN sample can contain aBN, tBN, and hBN). We also encourage the development of a standard or standards for porous BN materials which would allow for easier comparison between studies.

### Studying the Effect of Moisture

Many studies use porous BN in applications where moisture is present or where the reaction medium is water, and they omit evaluating the stability of the material upon usage. We recommend that researchers monitor any potential material decomposition. Analyses of the porosity, chemical composition, and crystalline patterns of the material before and after exposure/reaction will help in this regard.

### Forming Densified Porous BN

Today, there exist commercial scaled-up routes to producing hBN but not porous BN. Lab-scale studies on producing densified porous BN forms remain scarce and empirical. A better understanding and rationalization of the shaping of porous BN is needed to deploy the material. This knowledge gap is not specific to porous BN but extends to other emerging porous materials.

### Tuning Porous BN Chemistry and Structure

Studies have shown the possible structural and chemical tuning of porous BN and the impact on sorptive, storage, optoelectronic, and catalytic properties. Further work is needed to provide a set of design rules for porous BN, relating to doping, inclusion of defects, and porosity control. For instance, metal doping remains rare for porous BN but has proved effective for the catalytic and sorptive properties of other materials. We note that changing the chemistry/structure of porous BN can change its stability.

### Exploring the Most Relevant Applications

From a performance viewpoint, porous BN appears most relevant for gas storage and catalysis. For these fields, the material brings features that benchmark materials do not offer, i.e., combined porosity and inorganic nature with possible organic functionalization. For catalysis though, the actual role of BN as an intrinsic catalyst rather than a catalyst support must be better understood. For gas storage, the material’s high porosity and its possible metal functionalization make it an interesting platform deserving further investigation. For molecular separations, the advantage of porous BN over other adsorbents is less clear. Many adsorbents perform well, and their chemistry is easier to tune. The exception, though, includes using porous BN for high-pressure gas separation as porosity can drive the performance. In any case, porous BN’s water sensitivity must be addressed regardless of the targeted application.

### Testing Porous BN Consistently

BN materials are often tested for adsorption, catalysis, or storage under different experimental conditions, making direct comparisons difficult. This challenge is not specific to the material itself but to the application fields. This highlights the need to develop international standards providing measurement protocols to employ in adsorption, storage, and catalytic studies.

## Supporting Information Available

Tables containing detailed information and references about studies related to the doping and shaping of porous BN, as well as its applications in gas storage, molecular separations, and catalysis (PDF)
