# Supplementary material for: How to Tailor Porous Boron Nitride Properties for Applications in Interfacial Processes
Source: Acc Mater Res. 2023 Jan 30;4(2):143–55. doi: 10.1021/accountsmr.2c00148 (PMC9972479; doi:10.1021/accountsmr.2c00148)
Supplement: Supplementary file 1 — mr2c00148_si_001.pdf [file mr2c00148_si_001.pdf]

Supporting Information

How to tailor porous boron nitride properties for applications in interfacial processes

Ioanna Itskou, [1,¶] *Anouk L'Hermitte,*[1,2,¶] *Sofia Marchesini,*[3,¶] *Tian Tian,*[1,¶] *Camille Petit*[1*]

[1] Barrer Centre, Department of Chemical Engineering, Imperial College London, London SW7 2AZ, United Kingdom

[2] Department of Materials, Imperial College London, London SW7 2AZ, United Kingdom

[3] National Physical Laboratory, Hampton Road, Teddington, TW11 0LW, United Kingdom

[¶] Authors contributed equally to the work.

*Corresponding author: camille.petit@imperial.ac.uk

Table S1. Summary of studies relating to doping of porous boron nitride (BN) presented in Table 1.

Dopant	BN structure	Dopant content	Dopant chemical environment	Dopant role in the study	Nature of the study	Ref.
C	Porous BN	5 - 20 wt% C	Within basal planes	Reduction of bandgap	Experimental	[1]
C	Porous BN	10 -12 wt% C	Within basal planes	Addition of sorption sites	Experimental	[2]
C	BN nanosheets	8 - 15 wt% C	Within basal planes	Addition of sorption sites<br>Reduction of bandgap	Experimental	[3]
C	BN nanosheets	Approx. 6 - 9 at% C	Within basal planes	Addition of active catalytic sites<br>Reduction of bandgap	Experimental	[4]
C	BN nanosheets	14 - 23 at% C	Within basal planes	Addition of sorption sites	Computational + Experimental	[5]
O	BN nanosheets	5 - 20 at% O	Within basal planes<br>On edges	Reduction of bandgap<br>Tuning of magnetic properties	Computational+ Experimental	[6]
O	Porous BN	-	Within basal planes	Tuning of magnetic properties	Computational + Experimental	[7]
O	Porous BN	Only at% of B and N atoms included	Within basal planes	Reduction of bandgap	Experimental	[8]
C, O	Porous BN	8 at% C<br>6 at% O	Within basal planes	Increase in specific surface area and adsorption capacity	Experimental	[9]
Si	BN nanotubes	-	Within basal planes	New synthesis technique	Experimental	[10]
Si	BN nanotubes	0.08 at% Si experimental, 5 at% Si computational	Within basal planes	Reduction of bandgap	Computational+ Experimental	[11]
P	Porous BN	1 - 5 wt% P	Within basal planes	Addition of sorption sites	Experimental	[12]
S	h-BN	-	Within basal planes	Reduction of bandgap	Computational	[13]
S	h-BN	-	Within basal planes	Reduction in electrical resistivity	Experimental	[14]
F, C, O	Porous BN	-	Within basal planes	Reduction of bandgap	Computational	[15]

				Addition of sorption and catalytic sites		
P, S, O, F, Cl	h-BN monolayer	-	Within basal planes	Different reduction of bandgap depending on dopant	Computational	[16]
Cu	BN nanobelts	1 - 4 wt% Cu	Within basal planes	Creation of electric field within material<br>Electron transfer between reactant and Cu sites	Experimental	[17]
Sc, Ti, V, Cr, Mn, Fe, Co, Ni, Cu, Zn, Mo, Ru, Rh, Pd, Ag	h-BN monolayer	-	On monolayer surface	Reduction of reaction limiting potential	Computational	[18]
Pt, Pd, Au, Ag	BN monolayer	-	Within and on monolayer surface	Reduction of bandgap<br>Tuning of magnetic properties	Computational	[19]
Au	Porous BN	-	Composite form	Introduction of plasmonic heating	Experimental	[20]

Table S2. Summary of bottom-up methods to produce shaped porous BN along with the material properties.

**Materials**	**Precursors**	**Reaction conditions**	**S_{BET} [m^2 g^{-1}]**	**ρ^a [mg cm^{-3}]**	**Ref.**
BN aerogel	Graphene, boron oxide	1600-1800 °C (N_2)	440	60	[21,22]
BN aerogel	Graphene/carbon nanotube, borazine	900 °C (Ar/H_2); etching 600 °C	180-1450	0.6-4.6	[23]
BN aerogel	Graphene aerogel, borazine	1500 °C (Ar); etching 600 °C	1080	0.1	[24]
BN aerogel	Graphene oxide/carbon nanotube, boron oxide	1600 °C (N_2)	720	N/A	[25]
BN aerogel	N-doped graphite, boron oxide	1700 °C (N_2)	170	30.4	[26]
BN aerogel	Polymeric aerogel, boric acid	1000-1200 °C (NH_3)	1150-1410	180	[27]
BN aerogel	M·2B aerogel	1100 °C (NH_3)	780	15	[28]
BN aerogel	M·2B aerogel	1200 °C (NH_3)	920	15.5	[29]
BN aerogel	M·2B aerogel	1400 °C (Ar)	990	86.4	[30]
BN aerogel	M·2B pellet	900-1350 °C (N_2)	720-1030	154	[31]
BN aerogel	BNNS, 1,4-butanediol diglycidyl ether	RT aqueous reaction (24 h)	N/A	10.4-30	[32]
BN aerogel	BNNS, urea	RT aqueous reaction (1 week)	280	1.4-20	[33]
Structured BN	Porous BN powder	Spark plasma sintering	430	786	[34]
Structured BN	Boric acid-cured melamine resin	1000 °C (NH_3)	1530	310	[35]

[a] ρ = bulk density of shaped BN

Table S3. Summary of separation studies using porous BN as adsorbent, showing the type of separations (split into liquid/vapor and gas phase), example of species involved, adsorption conditions (initial concentration of pollutant (C_0)), quantity adsorbed (maximum adsorbed amount (q_{max})), and sorption mechanisms suggested (related to Table 3).

**Separation in liquid/vapor phase**	**Example species**	**C_0 range studied (mg L^{-1})**	**q_{max} range (mg g^{-1})**	**Sorption mechanisms suggested**	**Ref.**
Trace metal removal from water	Cu^{2+}	200 - 1870	200 - 819	Mainly ion-exchange interactions dominated by electrostatic and surface complexation. Hydrogen bonding and physisorption also reported. Increased adsorption in the presence of defects and higher SSA	[36,37]
	Pb^{2+}	6 - 200	204 - 845		
	Cr^{3+}	52 - 100	120 - 387		
	Ce^{3+}	52	282		
	Ni^{2+}	52 - 700	95 - 235		
	Co^{2+}	52	215		
	Cd^{2+}	200 - 600	107- 561		
	As^{5+}	1 - 50	5 - 32		
Removal of dyes from water	Methylene blue	10 - 350	107 - 13973	Mainly π-π stacking interactions. Electrostatic interactions, physisorption	[36–38]
	Methyl Orange	40	298 - 395		
	Rhodamine B	3 - 100	76 - 992		
	Basic Yellow	90	424		
	Congo Red	50 - 130	307 - 782		
PFAS from water	PFOS	200	~57	Electrostatic interactions	[39]
	PFDA	50	~90		
Oils and organic solvents	Engine oil	--	18000 - 56800	Capillarity effect, pore filling, SSA	[40–42]
	Ethanol	--	14000 - 25000		
	Acetone	--	25000		
	Toluene	--	16000 - 32000		
	Toluene/n-heptane/methanol	--	~600		
	Cyclohexane	--	5000		
Removal of bio waste from water	Tetracyclines	20 - 300	118 - 1100	π-π stacking interactions, electrostatic interactions, van der Waals forces	[36–38]
	Levofloxacin	20 - 300	318		
	Sulfamethazine	20 - 300	19		
Desulfurization of oil	Dibenzothiophene (DBT) in n-octane	500 - 800	35 - 65 mgS g^{-1}	Lewis acid-base interactions	[43,44]
Iodine removal	Iodine vapor	--	2120	Physisorption, Lewis acid-base interactions	[45]
	Iodine/ n-hexane	50	61		

**Separation in gas phase**	**Species involved**	**Conditions**		**q_{max} range (mmol g^{-1})**	**Sorption mechanisms suggested**	**Ref.**
		**T (°C)**	**P (bar)**			
Pre-combustion carbon capture	CO_2/H_2	25<br>25	20<br>40	CO_2: ~8<br>CO_2: ~19	Physisorption. Pore characteristics and electrostatic interactions key at low pressure. At high pressure volume and SSA play key role	[2,46]
Post-combustion carbon capture	CO_2/N_2	25	1	CO_2: 1.6 – 4.6<br>N_2: 0.2 – 0.3		

Natural gas sweetening	CO_2/CH_4	25<br><br>25	1<br><br>70	CO_2: 1.6 – 3.9<br>CH_4: 0.6 – 0.7<br>CH_4: ~9.0	Physisorption. Electrostatic interactions affect less CH_4	[2,46,47]
Formaldehyde from air	HCHO	25	1	0.63<br>(20 ppm C_0)	Physisorption, hydrogen bonding, π-π conjugation	[48]
Light hydrocarbons separation	N_2, CH_4, C_2H_4, C_2H_6, C_3H_6, C_3H_8	25	1	N_2: 0.1<br>CH_4: 0.5<br>C_2H_4: 2.1 C_2H_6: 3.0<br>C_3H_6: 4.5<br>C_3H_8: 5.5	Pure physisorption (dispersion forces)	[49]
Ammonia	NH_3	25	1	NH_3: 5.3 CO_2: 0.9	Physisorption, Lewis acid-base interactions and H-bonding	[50]

Table S4. Summary of gas storage studies using porous BN as adsorbent, showing the surface area of the material, and the performance metrics at given conditions (related to Table 4).

Gas stored	SSA_{BET} ($m^2\ g^{-1}$)	T (°C)	P (MPa)	Gas uptake (wt%)	Desorption level (%)[a]	Ref
H_2	150	RT[b]	10	1.8	30	51,52
	210	RT	10	2.6	30	51,52
	260	RT	10	2.9	20	52
	790	RT	10	4.2	50	53
	1560	-196	0.1	1.07	N/A	54
	215	25	10	4.07	N/A	55
	1150	-196	1	2.3	100	56
	1690	25	3	5.6	84	57
	1040-1070	-196	0.1	1.41-1.6	N/A	58
			1	1.9-2.14	N/A	
	1900	-196	1	2.6	100	59
	540	RT	5	5.7	89	60
	400-1290	-196	0.1	0.98-1.35	N/A	61
			1	1.65-2.33	100	
CH_4	440-720	0	0.1	0-3	N/A	62
	1050	0	0.1	1.1	N/A	47
	1500	25	7	13.6	N/A	35

[a] Percentage of gas released when the pressure is reduced to ambient condition; [b]RT stands for room temperature.

Table S5. Summary of catalysis studies using porous BN as a catalyst, showing the type of catalysis (i.e., heterogenous catalysis, photocatalysis or electrocatalysis), the targeted reaction and some of the key performance indicators (related to Table 5).

**Heterogeneous catalysis**						
**Reaction studied**	**Reaction conditions**	**Feed ratio/reactant mixture**	**Conversion rate**	**Selectivity rate**	**Catalyst stability**	**Ref.**
Oxidative dehydrogenation of propane	T = 490 °C	2:1:3.67 propane:O_2:N_2	14.0% propane	79% propylene	Min 32 h	63
Oxidative dehydrogenation of n-butane, isobutane	T = 460 °C	1:1:8 alkane:O_2:N_2	7.2% n-butane	76.2% butene	-	64
			6.2% isobutane	75.4% butene	-	
Methane oxidation	T = 690 °C	2:1:4 methane:O_2:N_2	20.5% methane	76% CO	Min 20 h	65
	T = 720 °C		37.0% methane	81.4% CO	-	
Styrene epoxidation	T = 80 °C	26.5 mmol TBHP 25 mL acetonitrile 10 mmol styrene	17.7-23.9% styrene	76.2-88.0% styrene oxide	Min 9 h	66
Aerobic oxidative desulfurization	T = 150 °C ambient pressure	40 mL oil (500 ppm DBT)	-	84.8-98.8% DBT removal	Min 10 recycling cycles	67
Acetylene hydrochlorination	T = 200 °C	1:1 HCl:acetylene	72% acetylene	> 96% vinyl chloride	-	68
Acetylene hydrochlorination	T = 280 °C	1:2 HCl:acetylene	>99% acetylene	> 99% vinyl chloride	Min 1000 h	
**Photocatalysis**						
**Reaction**	**Reaction phase**	**Irradiation range**	**Light source**	**Sacrificial agent**	**Production rate**	**Ref.**
H_2 generation	Liquid	200-2500 nm	300 W Xe arc lamp	TEOA	47.1 µmol H_2 $g^{-1}.h^{-1}$	69
H_2 generation	Liquid	Specified as UV-Vis (300-800 nm)	300 W Xe lamp	TEA	~45.0 µmol H_2 h^{-1}	1
		> 420 nm		TEA	3.6 µmol H_2 h^{-1}	
CO_2 photoreduction	Gas	> 420 nm		H_2O	4.7 µmol CO h^{-1} 1.5 µmol H_2 h^{-1}	
CO_2 photoreduction	Gas	> 325 nm	300 W Xe arc lamp	H_2	1.2 µmol CO g^{-1} h^{-1}	70
		> 400 nm			0.2 µmol CO g^{-1} h^{-1}	
CO_2 photoreduction	Gas	Specified as UV-vis	300 W Xe lamp	H_2O	12.5 µmol CO g^{-1} h^{-1} 3.3 µmol H_2 g^{-1} h^{-1}	71
**Electrocatalysis**						
**Reaction**	**Catalyst**	**Formation rate**	**Solution**	**Faradic efficiency/ Electron transfer number**	**Time/ Durability**	**Ref.**

N_2 fixation to NH_3	h-BNNS	22.4 µg NH_3 h^{-1} mg_{cat}^{-1}	0.1 M HCl	4.7% @-0.75V vs RHE	2 h	[72]
N_2 fixation to NH_3	Porous BN	18.2 µg NH_3 h^{-1} mg_{cat}^{-1}	0.1 M Na_2SO_4	5.5% @-0.70V vs RHE	2 h	[73]
Oxygen reduction reaction	Pt/porous BN	< 0.6% H_2O_2	0.1 M $HClO_4$	>3.99 electrons	10^4 cycles	[74]
Oxygen reduction reaction	BNNT<br>BNNS<br>sputter-deposited BN	-	0.5 M H_2SO_2	-	-	[75]

References

(1) Huang, C.; Chen, C.; Zhang, M.; Lin, L.; Ye, X.; Lin, S.; Antonietti, M.; Wang, X. Carbon-Doped BN Nanosheets for Metal-Free Photoredox Catalysis. *Nat. Commun.* **2015**, *6*. https://doi.org/10.1038/ncomms8698.

(2) Chen, S.; Li, P.; Xu, S.; Pan, X.; Fu, Q.; Bao, X. Carbon Doping of Hexagonal Boron Nitride Porous Materials toward CO2 Capture. *J. Mater. Chem. A* **2018**, *6* (4), 1832–1839. https://doi.org/10.1039/C7TA08515J.

(3) Chen, L.; Zhou, M.; Luo, Z.; Wakeel, M.; Asiri, A. M.; Wang, X. Template-Free Synthesis of Carbon-Doped Boron Nitride Nanosheets for Enhanced Photocatalytic Hydrogen Evolution. *Appl. Catal. B Environ.* **2019**, *241*, 246–255. https://doi.org/https://doi.org/10.1016/j.apcatb.2018.09.034.

(4) Fang, Y.; Merenkov, I. S.; Li, X.; Xu, J.; Lin, S.; Kosinova, M. L.; Wang, X. Vertically Aligned 2D Carbon Doped Boron Nitride Nanofilms for Photoelectrochemical Water Oxidation. *J. Mater. Chem. A* **2020**, *8* (26), 13059–13064. https://doi.org/10.1039/D0TA04593D.

(5) Li, H.; Zhu, S.; Zhang, M.; Wu, P.; Pang, J.; Zhu, W.; Jiang, W.; Li, H. Tuning the Chemical Hardness of Boron Nitride Nanosheets by Doping Carbon for Enhanced Adsorption Capacity. *ACS Omega* **2017**, *2* (9), 5385–5394. https://doi.org/10.1021/acsomega.7b00795.

(6) Weng, Q.; Kvashnin, D. G.; Wang, X.; Cretu, O.; Yang, Y.; Zhou, M.; Zhang, C.; Tang, D.-M.; Sorokin, P. B.; Bando, Y.; Golberg, D. Tuning of the Optical, Electronic, and Magnetic Properties of Boron Nitride Nanosheets with Oxygen Doping and Functionalization. *Adv. Mater.* **2017**, *29* (28), 1700695. https://doi.org/https://doi.org/10.1002/adma.201700695.

(7) Lu, Q.; Zhao, Q.; Yang, T.; Zhai, C.; Wang, D.; Zhang, M. Preparation of Boron Nitride Nanoparticles with Oxygen Doping and a Study of Their Room-Temperature Ferromagnetism. *ACS Appl. Mater. Interfaces* **2018**, *10* (15), 12947–12953. https://doi.org/10.1021/acsami.7b17932.

(8) Sevak Singh, R.; Yingjie Tay, R.; Leong Chow, W.; Hon Tsang, S.; Mallick, G.; Tong Teo, E. H. Band Gap Effects of Hexagonal Boron Nitride Using Oxygen Plasma. *Appl. Phys. Lett.* **2014**, *104* (16), 163101. https://doi.org/10.1063/1.4872318.

(9) Liu, H.; Li, P.; Wang, Y.; Wang, H.; Fang, Y.; Guo, Z.; Huang, Y.; Lin, J.; Hu, L.; Tang, C.; Liu, Z. Eco-Green C, O Co-Doped Porous BN Adsorbent for Aqueous Solution with Superior Adsorption Efficiency and Selectivity. *Chemosphere* **2022**, *288*, 132520. https://doi.org/https://doi.org/10.1016/j.chemosphere.2021.132520.

(10) Fan, Y.; Wang, Y.; Lou, J.; Xu, S.; Zhang, L.; An, L.; Heinrich, H. Formation of Silicon-Doped Boron Nitride Bamboo Structures Via Pyrolysis of a Polymeric Precursor. *J. Am. Ceram. Soc.* **2006**, *89* (2), 740–742. https://doi.org/https://doi.org/10.1111/j.1551-2916.2005.00761.x.

(11) Cho, Y. J.; Kim, C. H.; Kim, H. S.; Park, J.; Choi, H. C.; Shin, H.-J.; Gao, G.; Kang, H. S. Electronic Structure of Si-Doped BN Nanotubes Using X-Ray Photoelectron Spectroscopy and First-Principles Calculation. *Chem. Mater.* **2009**, *21* (1), 136–143. https://doi.org/10.1021/cm802559m.

(12) Li, Y.; Li, H.; Li, R.; Su, X.; Shen, S. Preparation of Phosphorus-Doped Boron Nitride and Its Adsorption of Heavy Metals from Flue Gas. *R. Soc. Open Sci.* **2022**, *7* (8), 200079. https://doi.org/10.1098/rsos.200079.

(13) Liu, B.; Lee, J. Y.; Yan, S. Enhanced Catalytic Oxidation of CO on Sulfur-Doped Boron Nitride. *ChemNanoMat* **2020**, *6* (2), 223–231. https://doi.org/https://doi.org/10.1002/cnma.201900516.

(14) Sugino, T.; Tanioka, K.; Kawasaki, S.; Shirafuji, J. Characterization and Field Emission of Sulfur-

Doped Boron Nitride Synthesized by Plasma-Assisted Chemical Vapor Deposition. *Jpn. J. Appl. Phys.* **1997**, *36* (Part 2, No. 4B), L463–L466. https://doi.org/10.1143/jjap.36.l463.

(15) Liu, Y.; Li, L.; Li, Q.; Lin, J.; Guo, Z.; Zhang, X.; Lu, Z.; Ma, Y.; Huang, Y.; Tang, C. Fluorine Doped Porous Boron Nitride for Efficient CO2 Capture and Separation: A DFT Study. *Appl. Surf. Sci.* **2021**, *556*, 149775. https://doi.org/https://doi.org/10.1016/j.apsusc.2021.149775.

(16) Asif, Q. U. A.; Hussain, A.; Nabi, A.; Tayyab, M.; Rafique, H. M. Computational Study of X-Doped Hexagonal Boron Nitride (h-BN): Structural and Electronic Properties (X = P, S, O, F, Cl). *J. Mol. Model.* **2021**, *27* (2), 31. https://doi.org/10.1007/s00894-020-04659-z.

(17) Li, L.; Hu, C.; Zhang, L.; Yu, G.; Lyu, L.; Li, F.; Jiang, N. Framework Cu-Doped Boron Nitride Nanobelts with Enhanced Internal Electric Field for Effective Fenton-like Removal of Organic Pollutants. *J. Mater. Chem. A* **2019**, *7* (12), 6946–6956. https://doi.org/10.1039/C9TA00255C.

(18) Cui, Q.; Qin, G.; Wang, W.; Sun, L.; Du, A.; Sun, Q. Mo-Doped Boron Nitride Monolayer as a Promising Single-Atom Electrocatalyst for CO(2) Conversion. *Beilstein J. Nanotechnol.* **2019**, *10*, 540–548. https://doi.org/10.3762/bjnano.10.55.

(19) Zhou, Y. G.; Yang, P.; Sun, X.; Wang, Z. G.; Zu, X. T.; Gao, F. First-Principles Study of the Noble Metal-Doped BN Layer. *J. Appl. Phys.* **2011**, *109* (8), 84308. https://doi.org/10.1063/1.3569725.

(20) Liang, C.; Zhang, Y.; Zhang, B.; Liu, X.-M.; Gao, G.-L.; Cao, J.; Xu, P. Plasmonic Heating-Promoted Photothermal Synthesis of α-Cyanoacrylonitriles Over Au/h-BN Catalysts. *Front. Chem.* 2021, 9, 732162. https://doi.org/10.3389/fchem.2021.732162

(21) Rousseas, M.; Goldstein, A. P.; Mickelson, W.; Worsley, M. A.; Woo, L.; Zettl, A. Synthesis of Highly Crystalline Sp2-Bonded Boron Nitride Aerogels. *ACS Nano* **2013**, *7* (10), 8540–8546. https://doi.org/10.1021/nn402452p.

(22) Pham, T.; Goldstein, A. P.; Lewicki, J. P.; Kucheyev, S. O.; Wang, C.; Russell, T. P.; Worsley, M. A.; Woo, L.; Mickelson, W.; Zettl, A. Nanoscale Structure and Superhydrophobicity of Sp2-Bonded Boron Nitride Aerogels. *Nanoscale* **2015**, *7* (23), 10449–10458. https://doi.org/10.1039/c5nr01672j.

(23) Song, Y.; Li, B.; Yang, S.; Ding, G.; Zhang, C.; Xie, X. Ultralight Boron Nitride Aerogels via Template-Assisted Chemical Vapor Deposition. *Sci. Rep.* **2015**, *5*, 1–9. https://doi.org/10.1038/srep10337.

(24) Xu, X.; Zhang, Q.; Hao, M.; Hu, Y.; Lin, Z.; Peng, L.; Wang, T.; Ren, X.; Wang, C.; Zhao, Z.; Wan, C.; Fei, H.; Wang, L.; Zhu, J.; Sun, H.; Chen, W.; Du, T.; Deng, B.; Cheng, G. J.; Shakir, I.; Dames, C.; Fisher, T. S.; Zhang, X.; Li, H.; Huang, Y.; Duan, X. Double-Negative-Index Ceramic Aerogels for Thermal Superinsulation. *Science* **2019**, *363* (6428), 723–727. https://doi.org/10.1126/science.aav7304.

(25) Kutty, R. G.; Sreejith, S.; Kong, X.; He, H.; Wang, H.; Lin, J.; Suenaga, K.; Lim, C. T.; Zhao, Y.; Ji, W.; Liu, Z. A Topologically Substituted Boron Nitride Hybrid Aerogel for Highly Selective CO2 Uptake. *Nano Res.* **2018**, *11* (12), 6325–6335. https://doi.org/10.1007/s12274-018-2156-z.

(26) Xue, Y.; Dai, P.; Zhou, M.; Wang, X.; Pakdel, A.; Zhang, C.; Weng, Q.; Takei, T.; Fu, X.; Popov, Z. I.; Sorokin, P. B.; Tang, C.; Shimamura, K.; Bando, Y.; Golberg, D. Multifunctional Superelastic Foam-Like Boron Nitride Nanotubular Cellular-Network Architectures. *ACS Nano* **2017**, *11* (1), 558–568. https://doi.org/10.1021/acsnano.6b06601.

(27) Xue, Y.; Dai, P.; Jiang, X.; Wang, X.; Zhang, C.; Tang, D.; Weng, Q.; Wang, X.; Pakdel, A.; Tang, C.; Bando, Y.; Golberg, D. Template-Free Synthesis of Boron Nitride Foam-like Porous Monoliths and Their High-End Applications in Water Purification. *J. Mater. Chem. A* **2015**, *4* (4), 1469–1478.

https://doi.org/10.1039/c5ta08134c.

(28) Lin, J.; Yuan, X.; Li, G.; Huang, Y.; Wang, W.; He, X.; Yu, C.; Fang, Y.; Liu, Z.; Tang, C. Self-Assembly of Porous Boron Nitride Microfibers into Ultralight Multifunctional Foams of Large Sizes. *ACS Appl. Mater. Interfaces* **2017**, *9* (51), 44732–44739. https://doi.org/10.1021/acsami.7b16198.

(29) Li, G.; Zhu, M.; Gong, W.; Du, R.; Eychmüller, A.; Li, T.; Lv, W.; Zhang, X. Boron Nitride Aerogels with Super-Flexibility Ranging from Liquid Nitrogen Temperature to 1000 °C. *Adv. Funct. Mater.* **2019**, *29* (20), 1–7. https://doi.org/10.1002/adfm.201900188.

(30) Wang, B.; Li, G.; Xu, L.; Liao, J.; Zhang, X. Nanoporous Boron Nitride Aerogel Film and Its Smart Composite with Phase Change Materials. *ACS Nano* **2020**, *14* (12), 16590–16599. https://doi.org/10.1021/acsnano.0c05931.

(31) Cao, C.; Yang, J.; Fu, K.; Zhai, Q.; Zhou, Z.; Ji, J.; Ma, Y.; Zhou, M.; Xue, Y.; Tang, C. Hierarchically Porous Boron Nitride Foams for Multifunctional Bulk Adsorbents. *Chem. Eng. J.* **2021**, *422*, 129896. https://doi.org/10.1016/j.cej.2021.129896.

(32) Zeng, X.; Ye, L.; Yu, S.; Sun, R.; Xu, J.; Wong, C. P. Facile Preparation of Superelastic and Ultralow Dielectric Boron Nitride Nanosheet Aerogels via Freeze-Casting Process. *Chem. Mater.* **2015**, *27* (17), 5849–5855. https://doi.org/10.1021/acs.chemmater.5b00505.

(33) Lei, W.; Mochalin, V. N.; Liu, D.; Qin, S.; Gogotsi, Y.; Chen, Y. Boron Nitride Colloidal Solutions, Ultralight Aerogels and Freestanding Membranes through One-Step Exfoliation and Functionalization. *Nat. Commun.* **2015**, *6*, 1–8. https://doi.org/10.1038/ncomms9849.

(34) Bernard, S.; Miele, P. Ordered Mesoporous Polymer-Derived Ceramics and Their Processing into Hierarchically Porous Boron Nitride and Silicoboron Carbonitride Monoliths. *New J. Chem.* **2014**, *38* (5), 1923–1931. https://doi.org/10.1039/c3nj01612a.

(35) Tian, T.; Hou, J.; Ansari, H.; Xiong, Y.; L'Hermitte, A.; Danaci, D.; Pini, R.; Petit, C. Mechanically Stable Structured Porous Boron Nitride with High Volumetric Adsorption Capacity. *J. Mater. Chem. A* **2021**, *9* (22), 13366–13373. https://doi.org/10.1039/d1ta02001c.

(36) Ihsanullah, I. Boron Nitride-Based Materials for Water Purification: Progress and Outlook. *Chemosphere* **2021**, *263*, 127970. https://doi.org/https://doi.org/10.1016/j.chemosphere.2020.127970.

(37) Yu, S.; Wang, X.; Pang, H.; Zhang, R.; Song, W.; … D. F.-C. E.; 2018, undefined. Boron Nitride-Based Materials for the Removal of Pollutants from Aqueous Solutions: A Review. *Elsevier*.

(38) Chao, Y.; Tang, B.; Luo, J.; Wu, P.; Tao, D.; Chang, H.; Chu, X.; Huang, Y.; Li, H.; Zhu, W. Hierarchical Porous Boron Nitride with Boron Vacancies for Improved Adsorption Performance to Antibiotics. *J. Colloid Interface Sci.* **2021**, *584*, 154–163. https://doi.org/10.1016/j.jcis.2020.09.075.

(39) Feng, Y.; Zhou, Y.; Lee, P. H.; Shih, K. Mineralization of Perfluorooctanesulfonate (PFOS) and Perfluorodecanoate (PFDA) from Aqueous Solution by Porous Hexagonal Boron Nitride: Adsorption Followed by Simultaneous Thermal Decomposition and Regeneration. *RSC Adv.* **2016**, *6* (114), 113773–113780. https://doi.org/10.1039/C6RA15564B.

(40) Gautam, C.; Tiwary, C.; Jose, S.; Brunetto, G.; … S. O.-A.; 2015, undefined. Synthesis of Low-Density, Carbon-Doped, Porous Hexagonal Boron Nitride Solids. *ACS Publ.* **2015**, *15* (12), 24. https://doi.org/10.1021/acsnano.5b05847.

(41) Marchesini, S.; Wang, X.; Petit, C. Porous Boron Nitride Materials: Influence of Structure,

Chemistry and Stability on the Adsorption of Organics. *Front. Chem.* **2019**, *7*, 1–9. https://doi.org/10.3389/fchem.2019.00160.

(42) Xue, Y.; Dai, P.; Zhou, M.; Wang, X.; Pakdel, A.; Zhang, C.; Weng, Q.; Takei, T.; Fu, X.; Popov, Z. I.; Sorokin, P. B.; Tang, C.; Shimamura, K.; Bando, Y.; Golberg, D. Multifunctional Superelastic Foam-Like Boron Nitride Nanotubular Cellular-Network Architectures. *ACS Nano* **2017**, *11* (1), 558–568. https://doi.org/10.1021/acsnano.6b06601.

(43) Xiong, J.; Yang, L.; Chao, Y.; Pang, J.; Zhang, M.; Zhu, W.; Li, H. Boron Nitride Mesoporous Nanowires with Doped Oxygen Atoms for the Remarkable Adsorption Desulfurization Performance from Fuels. *ACS Sustain. Chem. Eng.* **2016**, *4* (8), 4457–4464. https://doi.org/10.1021/ACSSUSCHEMENG.6B01156.

(44) Xiong, J.; Zhu, W.; Li, H.; Yang, L.; Chao, Y.; Wu, P.; Xun, S.; Jiang, W.; Zhang, M.; Li, H. Carbon-Doped Porous Boron Nitride: Metal-Free Adsorbents for Sulfur Removal from Fuels. *J. Mater. Chem. A* **2015**, *3* (24), 12738–12747. https://doi.org/10.1039/C5TA01346A.

(45) Li, G.; Huang, Y.; Lin, J.; Yu, C.; Liu, Z.; Fang, Y.; Xue, Y. E Ff Ective Capture and Reversible Storage of Iodine Using Foam-like Adsorbents Consisting of Porous Boron Nitride Micro Fi Bers. *Chem. Eng. J.* **2020**, *382*, 122833. https://doi.org/10.1016/j.cej.2019.122833.

(46) Marchesini, S.; McGilvery, C. M.; Bailey, J.; Petit, C. Template-Free Synthesis of Highly Porous Boron Nitride: Insights into Pore Network Design and Impact on Gas Sorption. *ACS Nano* **2017**, *11* (10), 10003–10011. https://doi.org/10.1021/acsnano.7b04219.

(47) Wang, D.; Xue, Y.; Wang, C.; Ji, J.; Zhou, Z.; Tang, C. Improved Capture of Carbon Dioxide and Methane via Adding Micropores within Porous Boron Nitride Fibers. *J. Mater. Sci.* **2019**, *54* (14), 10168–10178. https://doi.org/10.1007/S10853-019-03617-2.

(48) Ye, J.; Zhu, X.; Cheng, B.; Yu, J.; Jiang, C. Few-Layered Graphene-like Boron Nitride: A Highly Efficient Adsorbent for Indoor Formaldehyde Removal. *Environ. Sci. Technol. Lett.* **2017**, *4* (1), 20–25. https://doi.org/10.1021/acs.estlett.6b00426.

(49) Saha, D.; Orkoulas, G.; Yohannan, S.; Ho, H. C.; Cakmak, E.; Chen, J.; Ozcan, S. Nanoporous Boron Nitride as Exceptionally Thermally Stable Adsorbent: Role in Efficient Separation of Light Hydrocarbons. *ACS Appl. Mater. Interfaces* **2017**, *9* (16), 14506–14517. https://doi.org/10.1021/ACSAMI.7B01889.

(50) Yang, C.; Wang, J.; Chen, Y.; Liu, D.; Huang, S.; Lei, W. One-Step Template-Free Synthesis of 3D Functionalized Flower-like Boron Nitride Nanosheets for NH3 and CO2 Adsorption. *Nanoscale* **2018**, *10* (23), 10979–10985. https://doi.org/10.1039/C8NR02074D.

(51) Ma, R.; Bando, Y.; Zhu, H.; Sato, T.; Xu, C.; Wu, D. Hydrogen Uptake in Boron Nitride Nanotubes at Room Temperature. *J. Am. Chem. Soc.* **2002**, *124* (26), 7672–7673. https://doi.org/10.1021/ja026030e.

(52) Ma, R.; Bando, Y.; Sato, T.; Golberg, D.; Zhu, H.; Xu, C.; Wu, D. Synthesis of Boron Nitride Nanofibers and Measurement of Their Hydrogen Uptake Capacity. *Appl. Phys. Lett.* **2002**, *81* (27), 5225–5227. https://doi.org/10.1063/1.1534415.

(53) Tang, C.; Bando, Y.; Ding, X.; Qi, S.; Golberg, D. Catalyzed Collapse and Enhanced Hydrogen Storage of BN Nanotubes. *J. Am. Chem. Soc.* **2002**, *124* (49), 14550–14551. https://doi.org/10.1021/ja028051e.

(54) Portehault, D.; Giordano, C.; Gervais, C.; Senkovska, I.; Kaskel, S.; Sanchez, C.; Antonietti, M. High-Surface-Area Nanoporous Boron Carbon Nitrides for Hydrogen Storage. *Adv. Funct. Mater.*

2010, *20* (11), 1827–1833. https://doi.org/10.1002/adfm.201000281.

(55) Lian, G.; Zhang, X.; Zhang, S.; Liu, D.; Cui, D.; Wang, Q. Controlled Fabrication of Ultrathin-Shell BN Hollow Spheres with Excellent Performance in Hydrogen Storage and Wastewater Treatment. *Energy Environ. Sci.* **2012**, *5* (5), 7072–7080. https://doi.org/10.1039/c2ee03240f.

(56) Weng, Q.; Wang, X.; Zhi, C.; Bando, Y.; Golberg, D. Boron Nitride Porous Microbelts for Hydrogen Storage. *ACS Nano* **2013**, *7* (2), 1558–1565. https://doi.org/10.1021/nn305320v.

(57) Li, J.; Lin, J.; Xu, X.; Zhang, X.; Xue, Y.; Mi, J.; Mo, Z.; Fan, Y.; Hu, L.; Yang, X.; Zhang, J.; Meng, F.; Yuan, S.; Tang, C. Porous Boron Nitride with a High Surface Area: Hydrogen Storage and Water Treatment. *Nanotechnology* **2013**, *24* (15). https://doi.org/10.1088/0957-4484/24/15/155603.

(58) Weng, Q.; Wang, X.; Wang, X.; Liu, D.; Jiang, X.; Zhi, C.; Bando, Y.; Golberg, D. Preparation and Hydrogen Sorption Performances of BCNO Porous Microbelts with Ultra-Narrow and Tunable Pore Widths. *Chem. - An Asian J.* **2013**, *8* (12), 2936–2939. https://doi.org/10.1002/asia.201300940.

(59) Weng, Q.; Wang, X.; Bando, Y.; Golberg, D. One-Step Template-Free Synthesis of Highly Porous Boron Nitride Microsponges for Hydrogen Storage. *Adv. Energy Mater.* **2014**, *4* (7). https://doi.org/10.1002/aenm.201301525.

(60) Lei, W.; Zhang, H.; Wu, Y.; Zhang, B.; Liu, D.; Qin, S.; Liu, Z.; Liu, L.; Ma, Y.; Chen, Y. Oxygen-Doped Boron Nitride Nanosheets with Excellent Performance in Hydrogen Storage. *Nano Energy* **2014**, *6*, 219–224. https://doi.org/10.1016/j.nanoen.2014.04.004.

(61) Weng, Q.; Zeng, L.; Chen, Z.; Han, Y.; Jiang, K.; Bando, Y.; Golberg, D. Hydrogen Storage in Carbon and Oxygen Co-Doped Porous Boron Nitrides. *Adv. Funct. Mater.* **2021**, *31* (4), 7–9. https://doi.org/10.1002/adfm.202007381.

(62) Janik, J. F.; Ackerman, W. C.; Paine, R. T.; Hua, D.-W.; Maskara, A.; Smith, D. M. Boron Nitride as a Selective Gas Adsorbent. *Langmuir* **1994**, *10* (2), 514–518. https://doi.org/10.1021/la00014a029.

(63) Grant, J. T.; Carrero, C. A.; Goeltl, F.; Venegas, J.; Burt, S. P.; Specht, S. E.; McDermott, W. P.; Chieregato, A.; Hermans, I. Selective Oxidative Dehydrogenation of Propane to Propene Using Boron Nitride Catalysts. *Science.* **2016**, *354* (6319), 1570–1573. https://doi.org/10.1126/science.aaf7885.

(64) Venegas, J. M.; Grant, J. T.; McDermott, W. P.; Burt, S. P.; Micka, J.; Carrero, C. A.; Hermans, I. Selective Oxidation of N-Butane and Isobutane Catalyzed by Boron Nitride. *ChemCatChem* **2017**, *9* (12), 2118–2127. https://doi.org/https://doi.org/10.1002/cctc.201601686.

(65) Wang, Y.; Zhao, L.; Shi, L.; Sheng, J.; Zhang, W.; Cao, X.-M.; Hu, P.; Lu, A.-H. Methane Activation over a Boron Nitride Catalyst Driven by in Situ Formed Molecular Water. *Catal. Sci. Technol.* **2018**, *8* (8), 2051–2055. https://doi.org/10.1039/C8CY00163D.

(66) Fu, H.; Huang, K.; Yang, G.; Cao, Y.; Wang, H.; Peng, F.; Cai, X.; Gao, H.; Liao, Y.; Yu, H. Understanding the Catalytic Sites in Porous Hexagonal Boron Nitride for the Epoxidation of Styrene. *ACS Catal.* **2021**, *11* (14), 8872–8880. https://doi.org/10.1021/acscatal.1c02171.

(67) Wu, P.; Zhu, W.; Chao, Y.; Zhang, J.; Zhang, P.; Zhu, H.; Li, C.; Chen, Z.; Li, H.; Dai, S. A Template-Free Solvent-Mediated Synthesis of High Surface Area Boron Nitride Nanosheets for Aerobic Oxidative Desulfurization. *Chem. Commun.* **2016**, *52* (1), 144–147. https://doi.org/10.1039/C5CC07830J.

(68) Li, P.; Li, H.; Pan, X.; Tie, K.; Cui, T.; Ding, M.; Bao, X. Catalytically Active Boron Nitride in Acetylene Hydrochlorination. *ACS Catal.* **2017**, *7* (12), 8572–8577. https://doi.org/10.1021/acscatal.7b01877.

(69) Liu, Q.; Chen, C.; Du, M.; Wu, Y.; Ren, C.; Ding, K.; Song, M.; Huang, C. Porous Hexagonal Boron Nitride Sheets: Effect of Hydroxyl and Secondary Amino Groups on Photocatalytic Hydrogen Evolution. *ACS Appl. Nano Mater.* **2018**, *1* (9), 4566–4575. https://doi.org/10.1021/acsanm.8b00867.

(70) Shankar, R.; Sachs, M.; Francàs, L.; Lubert-Perquel, D.; Kerherve, G.; Regoutz, A.; Petit, C. Porous Boron Nitride for Combined CO2 Capture and Photoreduction. *J. Mater. Chem. A* **2019**, *7* (41), 23931–23940. https://doi.org/10.1039/c9ta02793a.

(71) Cao, Y.; Zhang, R.; Zhou, T.; Jin, S.; Huang, J.; Ye, L.; Huang, Z.; Wang, F.; Zhou, Y. B-O Bonds in Ultrathin Boron Nitride Nanosheets to Promote Photocatalytic Carbon Dioxide Conversion. *ACS Appl. Mater. Interfaces* **2020**, *12* (8), 9935–9943. https://doi.org/10.1021/acsami.9b21157.

(72) Zhang, Y.; Du, H.; Ma, Y.; Ji, L.; Guo, H.; Tian, Z.; Chen, H.; Huang, H.; Cui, G.; Asiri, A. M.; Qu, F.; Chen, L.; Sun, X. Hexagonal Boron Nitride Nanosheet for Effective Ambient N2 Fixation to NH3. *Nano Res.* **2019**, *12* (4), 919–924. https://doi.org/10.1007/s12274-019-2323-x.

(73) Zhao, J.; Ren, X.; Li, X.; Fan, D.; Sun, X.; Ma, H.; Wei, Q.; Wu, D. High-Performance N2-to-NH3 Fixation by a Metal-Free Electrocatalyst. *Nanoscale* **2019**, *11* (10), 4231–4235. https://doi.org/10.1039/C8NR10401H.

(74) Li, Q.; Li, L.; Yu, X.; Wu, X.; Xie, Z.; Wang, X.; Lu, Z.; Zhang, X.; Huang, Y.; Yang, X. Ultrafine Platinum Particles Anchored on Porous Boron Nitride Enabling Excellent Stability and Activity for Oxygen Reduction Reaction. *Chem. Eng. J.* **2020**, *399*, 125827. https://doi.org/10.1016/j.cej.2020.125827.

(75) Elumalai, G.; Noguchi, H.; Uosaki, K. Electrocatalytic Activity of Various Types of H-BN for the Oxygen Reduction Reaction. *Phys. Chem. Chem. Phys.* **2014**, *16* (27), 13755–13761. https://doi.org/10.1039/c4cp00402g.
